# Capability by Stacking: The Current Design Heuristic for Soft Robots

**DOI:** 10.3390/biomimetics3030016

**Published:** 2018-07-13

**Authors:** Stephen T. Mahon, Jamie O. Roberts, Mohammed E. Sayed, Derek Ho-Tak Chun, Simona Aracri, Ross M. McKenzie, Markus P. Nemitz, Adam A. Stokes

**Affiliations:** 1 School of Engineering, The Institute for Integrated Micro and Nano Systems, The University of Edinburgh, The King’s Buildings, Edinburgh EH9 3LJ, UK; s.mahon@ed.ac.uk (S.T.M.); s1686485@sms.ed.ac.uk (J.O.R.); m.mohammed@ed.ac.uk (M.E.S.); derek.chun@ed.ac.uk (D.H.-T.C.); simona.aracri@ed.ac.uk (S.A.); r.m.mckenzie@ed.ac.uk (R.M.M.); m.nemitz@ed.ac.uk (M.P.N.); 2 Engineering and Physical Sciences Research Council (EPSRC) Centre for Doctoral Training (CDT) in Robotics and Autonomous Systems, School of Informatics, The University of Edinburgh, Edinburgh EH9 3LJ, UK; 3 Department of Computer Science and Engineering, University of Michigan, 2260 Hayward St. BBB3737, Ann Arbor, MI 48109, USA

**Keywords:** soft robots, stacking, hierarchy, functional blocks, complexity, capability, design

## Abstract

Soft robots are a new class of systems being developed and studied by robotics scientists. These systems have a diverse range of applications including sub-sea manipulation and rehabilitative robotics. In their current state of development, the prevalent paradigm for the control architecture in these systems is a one-to-one mapping of controller outputs to actuators. In this work, we define functional blocks as the physical implementation of some discrete behaviors, which are presented as a decomposition of the behavior of the soft robot. We also use the term ‘stacking’ as the ability to combine functional blocks to create a system that is more complex and has greater capability than the sum of its parts. By stacking functional blocks a system designer can increase the range of behaviors and the overall capability of the system. As the community continues to increase the capabilities of soft systems—by stacking more and more functional blocks—we will encounter a practical limit with the number of parallelized control lines. In this paper, we review 20 soft systems reported in the literature and we observe this trend of one-to-one mapping of control outputs to functional blocks. We also observe that stacking functional blocks results in systems that are increasingly capable of a diverse range of complex motions and behaviors, leading ultimately to systems that are capable of performing useful tasks. The design heuristic that we observe is one of increased capability by stacking simple units—a classic engineering approach. As we move towards more capability in soft robotic systems, and begin to reach practical limits in control, we predict that we will require increased amounts of autonomy in the system. The field of soft robotics is in its infancy, and as we move towards realizing the potential of this technology, we will need to develop design tools and control paradigms that allow us to handle the complexity in these stacked, non-linear systems.

## 1. Introduction

Soft robotics represents a change in thinking about the dominant materials and methods of fabrication used in the manufacture of robotic systems. The inherent compliance of soft materials and the manufacturable fabrication techniques that have been developed for soft robots mean that these new systems show promise for applications including: assistive robotics in biomedical application, human–robot interaction, and search-and-rescue. The prevalent research direction focuses on actuators, and the current design heuristic in soft robotics is to increase the capability of the system by adding more actuators. In this paper, we describe the current design approach in soft robotics—what we call ‘stacking of functional blocks’—and we discuss the limitations of this approach.

By designing a functional block and then stacking these blocks, in a bottom-up design approach, one can quickly create higher-level functionality in a soft robot. As the community moves towards more capability in soft robotic systems, and we begin to reach practical limits in control, we predict that we will require increased amounts of autonomy in the system by moving from a one-to-one mapping of functional blocks to outputs from control hardware to having more functional blocks with fewer control outputs.

Here, we begin to formalize a framework, and we start to explore how we, as a community, can begin to develop tools to allow our designers to build more complex, and more useful soft robotic systems.

### 1.1. A New Class of Systems

Conventional robots are extensively used in the manufacturing industry to perform well-defined tasks. Robots made of ‘hard’ materials lack the compliance that is required for human–robot interactions; they are built from rigid links and joints. Soft robots, in contrast, are built from soft materials such as silicone rubbers which enable continuous deformation, and enable a large range of motion. Due to their nearly infinite degrees of freedom, soft robots can achieve motions similar to biological systems. Soft systems are able to operate in hostile or poorly accessible environments such as rough and unstructured terrains [1,2], or confined spaces [3], whilst simultaneously allowing compliance for safe interactions.

The enabling technology of soft robotics is, primarily, the soft materials used to fabricate the body and actuators. These robots are characterized as ‘soft’ by their intrinsic Young’s modulus, an extrinsic elasticity, functions of the intrinsic material property, and material geometry. Soft systems—in comparison to hard materials—have a global modulus which is much closer to that of biological systems. Soft robots, therefore, have been defined by Rus and Tolley as systems with a Young’s modulus in the range of (soft) biological materials and which are capable of autonomous behaviors [4]. This emulation of biology is a major inspiration in soft robotics for actuation methods [5,6,7], adaptive behaviors [8,9,10,11], and locomotion [3,12,13].

The control and movement of rigid bodies can be generally described by three degrees of freedom where the kinematics and dynamics of the system are well defined. In contrast, due to the nearly infinite degrees of freedom that arise from material deformations, describing motion and developing control are significant challenges for soft robotic systems.

### 1.2. Innovation in Traditional Fabrication Techniques

Soft robots often contain complex internal geometries that take advantage of the large deformations provided by soft materials. Researchers have used additive manufacturing and soft lithography to manufacture complex soft structures [14]. Additive manufacturing is an umbrella term that refers to technologies that build three-dimensional (3D) structures by adding layer-upon-layer of materials such as 3D printing. These technologies are used to fabricate molds; the blueprint of soft robotic systems. There are even more sophisticated additive manufacturing technologies which allow simultaneous deposition of multiple materials. The Octobot is an excellent example of multi-layer fabrication; the entire robot, including its chemical catalysts and actuators, are fabricated in a single process [15]. Additive manufacturing also enables inline computational design and verification processes, which could significantly improve and streamline fabrication processes. This fabrication method is different to techniques that are being used in high-throughput productions. In conventional robotics, the design methodology is based on a ‘simulate—build—test’ loop with an emphasis on the simulation. A hard robot usually consists of well-defined components and rigid links. This type of robot can be simulated by defining the Jacobian of the system and then applying well-established methodologies, such as inverse kinematics.

In soft robotics we often use composites of heterogeneous materials, of which many are yet to be completely characterized. The testing of a newly developed soft robotic system is paramount since soft materials often lead to unpredictable deformations during actuation. Finite element models can be used to simulate soft systems. Whereas static soft systems can be simulated very accurately, dynamic models suffer from computational expense [16]. Fortunately, soft robotic materials are usually low-cost and the turnaround time between a finished design and a fabricated soft robot only takes a couple of days. As a result, it is common that designers of soft robots use empirical and Edisonian design loops in which the designer cycles between building and testing a system.

#### 1.2.1. Design Embodiment of Soft Robotics

The continuum behavior of a robot can be described when the shape and movement of the robot is defined by a continuous function. Generally, a robot has enough discrete links to give the minimum number of degrees of freedom necessary to perform a task. If the robot is designed with more than the minimum number of degrees of freedom then this system can be described as kinematically redundant. The links in a soft robot are molecular giving a nearly infinite number of degrees of freedom. The continuum arm in soft robots is inspired by examples found in nature such as an elephant’s trunk [17], caterpillars [18], and octopus’ arms [8]. These systems allow a vast number of degrees of freedom and for such complex systems, geometric approximations through constant curvature and machine learning can be used for control [19,20]. The use of a neural network or other machine learning techniques turns the hyper redundant system into a model-free statistical system. In general, continuum robots use an external observation sensing modality, such as motion tracking, for control. For example, there are several major challenges for using continuum robots in medical applications such as lack of sensing, control, and human–robot interaction according to Burgner-Kahrs et al. [21].

#### 1.2.2. Untethered Control in Soft Systems

In their most recent work, Rich et al. [22] provide a comprehensive overview of untethered soft robots. Tolley et al. [1] developed a pneumatically powered untethered soft robot with embedded air compressors, batteries, valves, and controllers. Their soft robot demonstrated resilience to extreme environmental conditions. The speed and mobility of their soft robot was enabled by expansion of the soft materials; so they controlled the air flow-rate to actuate the pneumatic legs.

Underwater robots imitating aquatic animals such as fish [2,9,23], octopus [24], lamprey [25,26], and mantas [27] have shown promising demonstrations of untethered exploration. These robots use electrical control systems to regulate buoyancy, adjust dive planes, and stabilize movements.

Wehner et al. [15] have recently shown a fully integrated design and fabrication strategy for entirely soft autonomous robots using fluidic logic. The Octobot possesses an oscillator that regulates the fluid flow to an actuator providing a method of locomotion.

### 1.3. Diverse Applications of Soft Robotic Systems

#### 1.3.1. Exploration in Unstructured Environments

Soft robotics has used nature as a source of inspiration for developing robot locomotion in unstructured environments. Research has focused on understanding locomotion in nature to improve robot designs [9,11,13,28]. Conventional robots often fail in unstructured environments due to unexpected environmental changes such as slopes, dynamically moving objects, or human interactions. The material compliance of soft robots can potentially help encountering such unexpected environmental changes. For example, soft robots can squeeze into niches, absorb collisions, and survive falls.

Hawkes et al. [28] developed a soft robot that navigates by physically growing through constrained environments. This tethered robot uses pressure-driven lengthening from the tip and asymmetric lengthening as an active steering control. The Arthrobot [29] is a semisoft robot inspired by arthropods and arachnids. Arthrobots are built from hollow tubes, which are cut with a notch, and then fitted with an inflatable rubber balloon and elastomeric tendon. These multilegged robots are capable of walking up slopes and skimming across the surface of a pool of water. Katzschmann et al. [2] recently introduced a soft robotic fish (SoFi) and demonstrated its underwater locomotion, manoeuvrability, sensing, and communication capabilities. SoFi was designed for exploration tasks in aquatic environments and observation of marine life. It is the most recent embodiment of previous soft robotic fish prototypes [9,23].

#### 1.3.2. Biomedical Applications

Soft robots are ideal for human–robot interactions. Their materials are softer than the materials they interact with, which makes them inherently safe. Soft systems have been used in a series of biomedical applications. An implantable soft robotic sleeve has been used in targeted therapy for cardiac regeneration in ischemic heart disease to restore circulation in the heart and re-establish muscle function [30]. Vacuum-driven soft pneumatic actuators have been embedded into wearable human spine-assistive robotic devices [31,32]. Soft actuators have been embedded into wearable assistive technologies for hand, elbow, and stroke rehabilitation [33,34,35,36,37]. A modular soft-robotic system consisting of a soft robotic exoskeleton, a brain–machine interface, and a glove with embedded force sensors, has been used as a smart orthotic rehabilitation system [35].

### 1.4. Capability of Soft Systems

#### 1.4.1. Stacking of Functional Blocks

Soft robotics has the potential to be used in applications involving human–robot interaction, for example: biomedical devices and search-and-rescue scenarios. In this paper, we have decomposed the behavior of some soft robots into a group of functions. These functions are representations of defined physical modules which can be embodied as functions of mechanical effort and flow. We define a new term, ‘functional blocks’, as those physical modules which satisfy the minimum behavior necessary, as given by the functional decomposition of the task. By abstraction, this block does not have defined physical properties, however it must have a form of implementation to bring the conceptual hierarchies to physical meaning. The physical implementation of this functional block is considered as a ‘module’. The module is not itself part of the behavioral decomposition but, the result of its mechanical work done on the environment exhibits a behavior which is part of the behavioral decomposition. When referring to functional blocks in this paper, it is the resultant behavior of the mechanical work from these modules that is to be considered.

In this paper, we use the term ‘stacking’ as a flexible term denoting the ability to combine functional blocks to create a larger system while minimizing the number of control outputs. A soft system that uses stacked functional blocks will exhibit more complex behavior than a system which uses one single functional block. Stacking can be associated with the direction and repetition of functional blocks. In this paper, however, we do not intend the term ‘stacking’ to mean only the combination or repetition of similar functional blocks, and we do not consider the geometric direction of stacking.

The arrangement of functional blocks, in the soft robotic systems that we studied, is hierarchical in design and the system behavior emerges from multiple levels of abstraction. We see a trend in the literature to stack functional blocks to increase the capability of the system by showing a diverse range of complex motions. In this bottom-up approach, a component is designed, optimized, and then stacked together to create higher-capability systems with more complex behaviors.

#### 1.4.2. Emergence of Complex Behaviors

**Figure 1:**
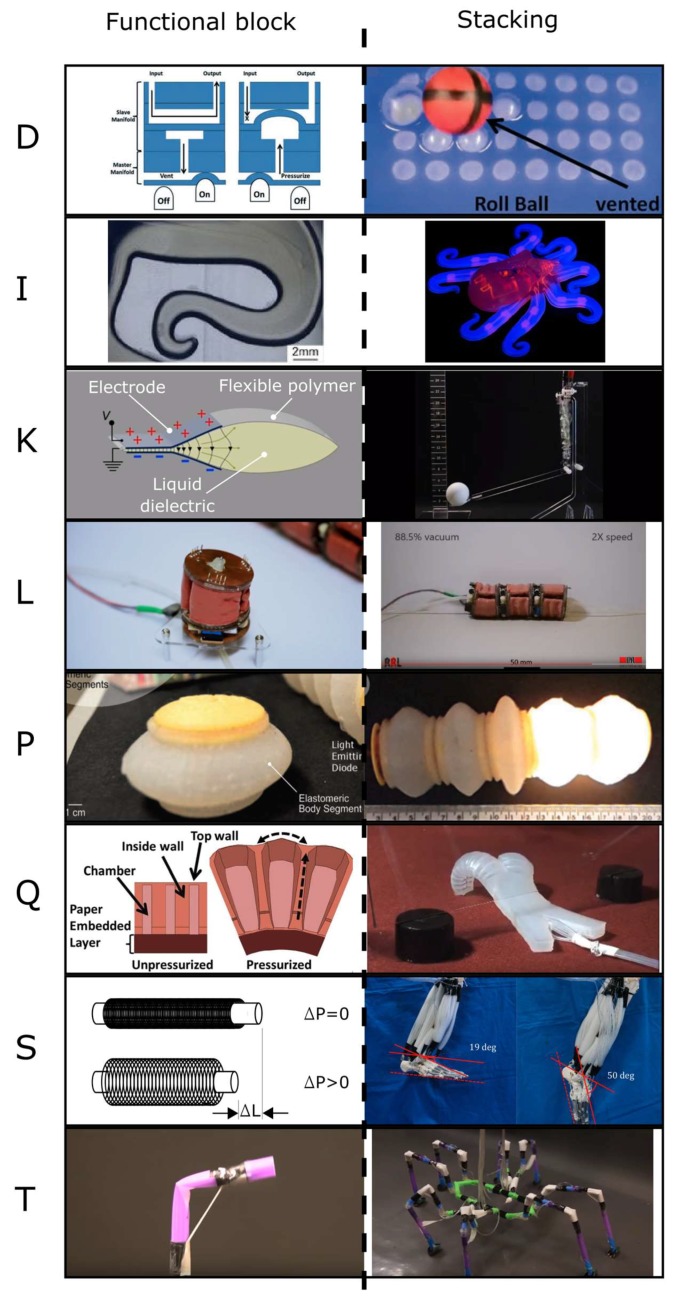
An example of functional blocks that are stacked to create systems that are greater than the sum of their parts. (**D**) A pneumatic Braille display. Reproduced from [38] with permission of The Royal Society of Chemistry; (**I**) the Octobot. Reprinted by permission from Springer Nature: Nature [15] (2016); (**K**) the Peano hydraulically amplified self-healing electrostatic actuator (HASEL) [39]; (**L**) the vacuum-powered soft pneumatic actuator (V-SPA) [31,32]; (**P**) the Wormbot. Wormbot [13] is licensed under CC BY 2.0 [40]; (**Q**) the multigait soft robot. Reproduced with permission from [3]; Copyright 2011 National Academy of Sciences; (**S**) McKibben actuators as a redundant musculoskeletal robot. Redundant musculoskeletal robot with thin McKibben muscles [41] is licensed under CC BY 4.0 [42]; (**T**) the Arthrobot [29].

The diverse range of applications of soft robots is mainly due to their capability to perform a variety of complex motions. The stacking of functional blocks to increase the capability of the system is a prevalent engineering approach; we observe this approach in many systems, as shown in Figure 1 and Table 1.

Mosadegh et al. [38] showed pneumatic inflation of small channels in an elastomeric material and stacked 32 independent actuators to control and roll a ball across the manifold. The Octobot actuates two clusters of four legs through a microfluidic soft controller from two fuel reservoirs [15]. The Peano hydraulically amplified self-healing electrostatic actuator (HASEL) actuator demonstrates muscle-like behavior by stacking three functional blocks (actuators) in series [39]. The vacuum-powered soft pneumatic actuator (V-SPA) is stacked in five configurations to demonstrate mobility, manipulation, interaction, and mechanical tuning [31,32]. The Wormbot is inspired by the earthworm and it consists of electromagnetic actuators that are stacked in series to demonstrate peristaltic motion [13]. The multigait soft robot has pneumatic actuators, which are stacked in parallel and it is capable of complex motions such as crawling and walking [3]. Kurumaya et al. [41] reported on a lower limb musculoskeletal robot which uses 20 multifilament muscles bundled from McKibben actuators.

All these systems demonstrate increased capability, that is to say the emergence of high-level behaviors, through the stacking of functional blocks.

### 1.5. Control Paradigm

**Figure 2:**
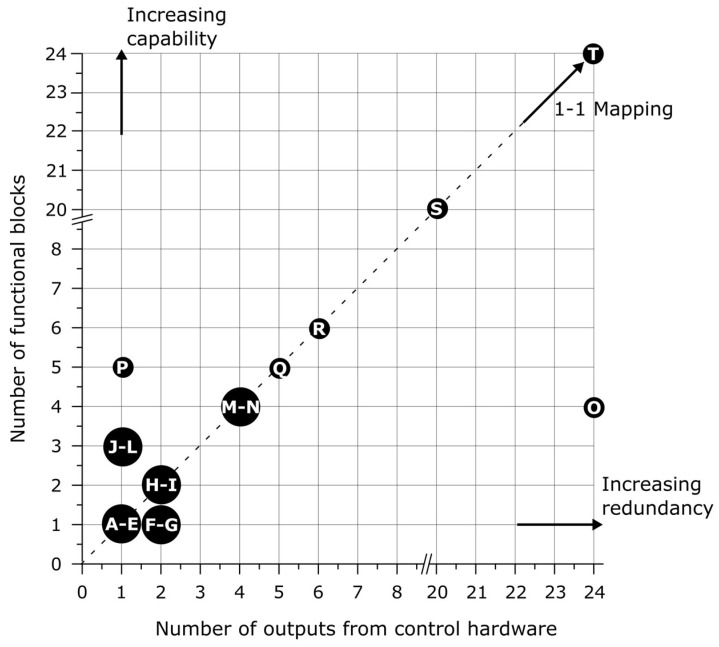
This graph illustrates a one to one mapping of functional blocks to outputs from control hardware in soft systems. There is a limit to the number of functional blocks in a system if each block has an independent control line. Reference: (**A**–**E**) Christianson et al. [43]; Keithly et al. [11]; Li et al. [44]; Mosadegh et al. [38]; Sareh et al. [45]; (**F**–**G**) Laschi et al. [8]; Stokes et al. [46]; (**H**–**I**) Katzschmann et al. [23]; Wehner et al. [15]; (**J**–**L**) Acome et al. [7]; Kellaris et al. [39]; Robertson and Paik [31]; (**M**–**N**) Bartlett et al. [47]; Mosadegh et al. [48]; (**O**) Wei et al. [12]; (**P**) Nemitz [13]; (**Q**) Shepherd et al. [3]; (**R**) Tolley et al. [1]; (**S**) Kurumaya et al. [41]; (**T**) Nemiroski et al. [29].

Figure 2 shows our review of 20 soft systems that increase capacity by adding more functional blocks. Here, we define the control of functional blocks as an output from some control hardware. The parameters for the construction of Figure 2 are described in Table 1. The trend is clear that there is a one-to-one mapping of outputs from control hardware to functional blocks. It is obvious that as we move towards more capability in soft robotic systems, continuing this trend, we will begin to reach practical limits in control due to size restrictions of pneumatic lines and pressure limitations across large pneumatic networks. As we add more functional blocks, we will hit a limit with the number of parallel control lines. We can label the limits of each axis: the more control outputs to a functional block, the more fine-tuned control or redundancy there is in the system; if there are more functional blocks than control lines, then the system has more capability. These concepts are illustrated by the redundant control on the soft pneumatic maggot bot [12] and the eight-legged Arthrobot [29], while the Peano-HASEL actuators [39], V-SPA [31], and Wormbot [13] show a wide range of motions and capabilities. We predict that as soft robotic systems increase in capability, this practical limit in control will move towards the upper left quadrant of Figure 2, and we will begin to see increased autonomy in soft systems.

**Figure 3:**
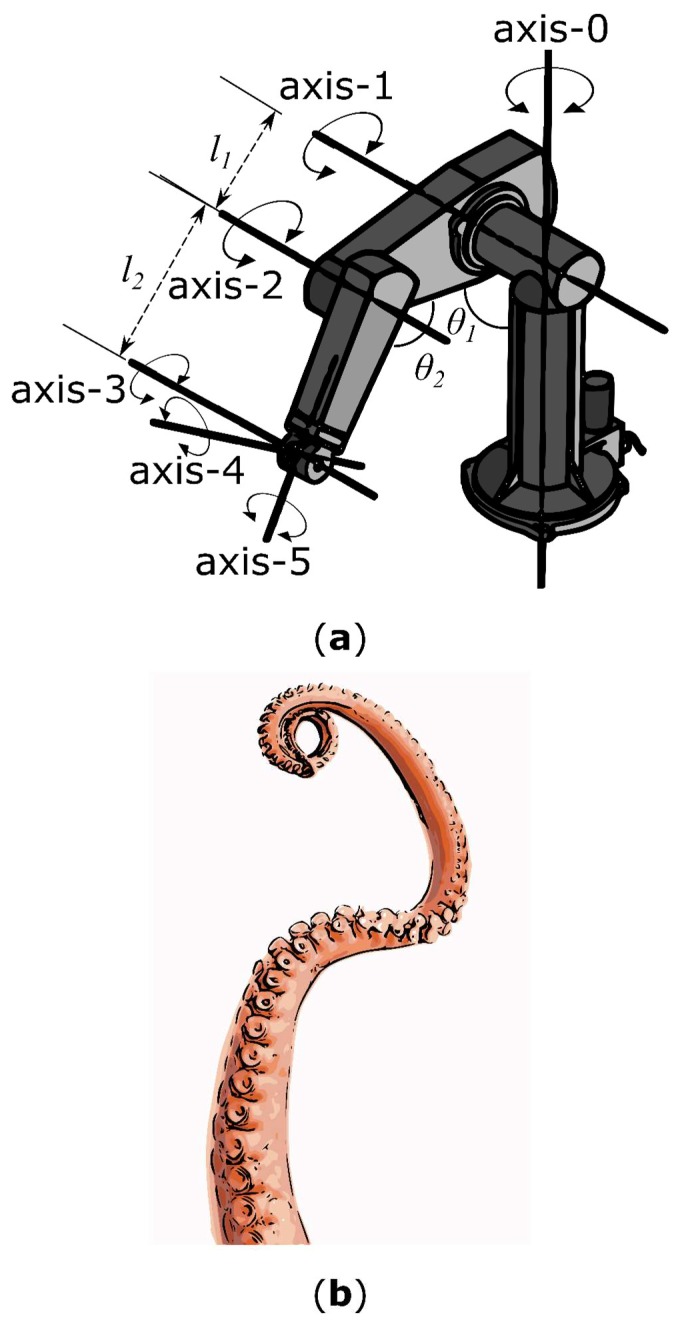
An illustration of a hard-bodied system and a soft-bodied system that could be employed to grasp and manipulate objects. (**a**) A simple rigid bodied system (Programmable Universal Machine for Assembly (PUMA) robot); (**b**) a complex soft-bodied system. The functionality of the PUMA robot can be broken down into (1) grasping an object, and (2) moving in free space. These functions can be further broken down until the system is described fully. The physical hierarchy of the PUMA robot (e.g., electric direct current (DC) servo motors, four-way pneumatic solenoid grippers, nuts and bolts, etc.) has little or no relevance to soft-bodied systems [49], which have more characteristics in common with an octopus arm. Both the PUMA robot and the octopus arm, however, have the same behavior—to grasp and manipulate an object—but each uses a completely different physical implementation.

Figure 3 shows a rigid link robotic arm and a soft, continuously deformable octopus arm. From the perspective of the physical implementation, the two systems are unrelated. However, from the functional, task-oriented view, the rigid link robot performs the same operations as the octopus arm—the gripping and manipulation of objects. The Programmable Universal Machine for Assembly (PUMA) robot has six degrees of freedom and requires six electric direct current (DC) servo motors and one four-way pneumatic solenoid gripper. The complex soft bodied system is capable of continuous deformation as the links in the system are molecular, giving a nearly infinite number of degrees of freedom. If we follow this one-to-one mapping of functional blocks to outputs from control hardware, as seen in Figure 2, then this type of hyper redundant system cannot be implemented using our existing methods. The number of control lines becomes prohibitively large. There are soft robotic systems which use arms that are inspired by the octopus; notably, Laschi et al. [8] focused on the broad arrangement of longitudinal and transverse muscles using cables and shape-memory alloy (SMA) springs. This innovative muscular hydrostat concept reduced the control of the system to only two cables, but the sacrifice was the capability of the arm to perform deterministic gripping and movement.

## 2. Stacking and Hierarchy as a Heuristic for Soft Robotic Design and Control

Soft robotics is currently limited in capability, in part due to the difficulty designers have working with the unknown and complex response of soft materials in their environment. The method proposed here aims to abstract this problem to a subset of discrete representations of defined physical modules which can be represented as functions of mechanical effort and flow. These physical modules must also function as the physical implementation of a subset of discrete behaviors which are presented as a decomposition of the desired task that the soft robot should perform. The method aims to provide a framework from which the designer can employ various techniques to combine and consolidate the modules into a working soft robot.

We have identified a top-down approach for the design of soft robots utilizing stacking of functional blocks to increase the capabilities of new systems. The steps involved in the stacking and hierarchy heuristic for the design and control of soft robots are: (1) defining the behavior and identifying the requirement for the task; (2) decomposing this behavior into a set of functions and further reducing to subfunctions the behavior has been fully described; (3) describing a functional block with the minimum behavior necessary with an associated effort and flow variable; (4) modeling the functional block to establish an empirical relationship if none already exist; and (5) stacking the functional blocks to progress to systems and behaviors.

This method relies on the fact that the design is task oriented and therefore everything about the nature of the task must be utilized and defined relative to the behavioral output of the machine. By capturing the behavioral decomposition and linking it to the physical modules mentioned above, both the design and control of the soft robot can be encompassed as part of an integrated design flow.

This method is hierarchical in its nature, with hierarchies comprising of a decomposition of both the behavioral and the physical systems. The goal of this method is to utilize stacking as method such that the global behavior of the finished soft robot is sufficiently more complex than the behavior of its individual modules. Therefore, the critical features of this method are a sufficiently descriptive behavioral decomposition coupled with an energetically sound physical module description. From this point, the methods by which they can be stacked are dependent on the solution. This approach draws on parallels with standard optimization procedures, as the design process can be set up to reward efficiency towards the first working solution or to explore the design space for increased novelty.

The purpose of this method is to allow the designer to better explore the problem space such that they can explore the potential solutions using only abstracted models of functional blocks. This approach would, potentially, decrease the number of iterations of functional blocks, and improve the creativity of soft robot designers by allowing them to focus on the behavior of the whole robot rather than on the modules, thereby reducing the time spent on designing a specific behavior.

### 2.1. Functional Decomposition as a Principle

When defining a hierarchical design principle such as this, it is important to ground the discussion in existing functional decomposition methods as they will form the structure around which our definitions will be defined. Functional decomposition serves as a mechanism by which often complex problem spaces can be divided into hierarchies such that the design problem is simplified and streamlined.

The prominent approaches to functional modeling can be divided into two categories: (1) functional basis, or black box approaches, that trace flows through a system [50]; and (2) hierarchies of functions that alternate between functions and physical means, from systems to components [51,52].

**Figure 4:**
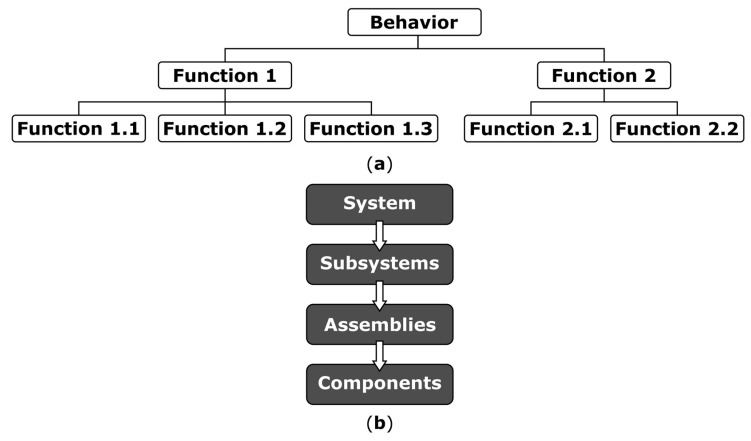
Hierarchical description of a system. (**a**) The behavior can be broken down into a hierarchy of functions, with each function comprising of subfunctions until the complete behavior of the system can be fully described. This is an important task and must be described fully as the behavioral description of the system. The functional hierarchy is a full description of the system without reference to technology. (**b**) The physical hierarchy describes how each function is implemented. The system is broken down into subsystems, and then into assemblies, and finally components. This top-down approach ensures that the task can be traced back to the behavior of the system. Both the physical hierarchy and the functional hierarchy describe the complete behavior of the system, but the descriptions are independent of each other as one describes the function and the other describes the technology.

A functional basis describes engineering design as a set of systematic and repeatable principles. Here, we decompose the behavior of soft robotic systems into physical hierarchy and functional hierarchy as described by Umeda and colleagues [51,52]. Example hierarchies are shown in Figure 4a,b. The aim of a hierarchy in design is to define tasks and to produce a system that matches the requirements of the behavior. The functional hierarchy describes the behavior of the system without reference to the technology, but instead focusing on the task to be fulfilled by each block. Physical hierarchy describes the system in terms of its assemblies, components, and parts. Breaking a system down into a functional and physical hierarchy focuses on defining needs of the system and required functionality early in the development. This approach can manage the expectations of the system without unnecessary functionality.

This idea is a common part of the conceptual design phase of systems engineering described by Pahl and Beitz [53]. The essence of task must be understood early in the design process, before the function structures are established, to safeguard the correct implementation of the needs of the system. This functional description translates the needs or behavior of the system into a sequenced and traceable hierarchy. The result is a hierarchy which details the requirements of the systems and the interfaces between subsystems.

A physical description of a system is related to the technology of the system. The description explains what the system elements are, what the elements look like, and how the elements are manufactured, integrated, and tested. The physical hierarchy takes a physical description and creates a top-level entity known as the system. The system comprises of subsystems, each subsystem comprising assemblies, with each assembly comprising of many components. The hierarchical description allows management of planning, design, and implementation of complex systems. The physical hierarchy is implemented after the functional hierarchy has been established. The upper-level trade-offs and feasibility are conducted before deciding on a physical implementation to ensure that the task of the behavior is always forefront and avoiding any unnecessary functionality. A complete physical hierarchy describes the system without context to the behavior of the system.

Both the functional and physical hierarchies fully describe the system independently of each other. The functional description describes the behavior while the physical description describes the technology of the system. However, since the functional description is a higher-level description of the system, the physical description and hierarchy can change rapidly with innovations in the technology.

### 2.2. Stacking Systems

In this paper, we use the word ‘stacking’ as a flexible term to denote a methodology to move between the functional and physical hierarchy; Figure 4 provides a reference on the nature of these hierarchies. A functional block has an accompanying module, and stacking describes the method that progresses modules-and-blocks to systems-and-behaviors. These methods could, in practice, range from analytical design methods to physical fabrication methods.

It is difficult to define the exact nature of these methods as they will alter on a case-specific basis and they are intrinsically linked to the quality of the behavioral and physical decomposition. The link between the functional block and its module will dictate the methods available to the designer. More flexibility in this manner will allow for an increase in the number of novel solutions, and a greater ability to explore the solution space of the design problem. Currently, the literature suggests that stacking is a process that occurs in fabrication and assembly, and this thinking essentially limits the capability of a designer to assemble modules in series, in parallel, or along a geometric theme that has taken inspiration from other sources, such as nature. It cannot always be the case that these solutions will always be fit for purpose when designing soft robots.

As previously mentioned, the quality of the behavioral and physical decomposition allows for more enhanced stacking methods to be employed. Ideally, mathematical and optimization methods would be employed such that features such as orthogonality, superposition, substitution, and aggregation can be induced analytically in the positioning and interactions of modules and functional blocks. These methods would begin to allow for the vast potential of ‘stacking’ to be unlocked. The word ‘stacking’ is intended to make the general design-concept in our paper accessible to the reader.

### 2.3. Modeling a Functional Block

A soft robot with a deformable actuator is difficult to model dynamically due to the nonlinear response of the soft materials. Despite this challenge, the design and the method of manufacture of soft robots is repeatable and consistent. In terms of a soft actuator as a physical module, the observed responses to external perturbations and stimuli should be consistent and should occur with a low variance across a range of the same manufactured module. In comparison with the development of steam-tables in thermodynamic engineering design, this pragmatic approach allows for statistically meaningful, empirical relationships to be drawn experimentally, leading to a repository of abstract models of modules which have inputs and outputs, and likely a model-free description in between.

The functional block can be described by an equation with ideal function plus losses due to unwanted expansions or other unwanted effects. The outputs and total losses will limit the stacking of blocks, but will provide a general framework to work within an existing functionality. Information flow can be an important indicator of input and output, another indicator of the relationships from input to output is Boolean algebra in a digital inspired approach. Automated rules for verification and validation of design can be devised so that the system of intermediate blocks needed to transform an input to a desired output can be built procedurally. With the definition of a functional block in mind, particularly its intrinsic duality with a physical module, it should be clear that the behavioral description of the design space is critically limited by the ability to model a physical module with enough accuracy and with sufficient information so that ‘stacking’ procedures can be applied to it.

Stone et al. [50] describe the decomposition strategy on a functional basis, or a black box approach. In their paper, a function is characterized in a verb–object format and is intended to comprehensively describe a mechanical design space providing clear definitions for each function and flow. The goal of that approach is to formulate the engineering design as a set of systematic and repeatable principles.

The analytical relationship between the inputs and outputs of a functional block can be determined experimentally, and will provide the abstraction of that functional block. The relationship between inputs and outputs of functional block satisfies the conservation laws of mass and energy. A continuity equation can ensure that thermodynamic laws are satisfied. These continuity variables can be determined experimentally and kept for sharing and reuse. The experimental analysis of soft robotic manipulators can provide coefficients to calibrate or reproduce a model state. Knowing the inputs and outputs in a black box function can allow for the rapid design of a stacked functional system using conservation as verification to ensure validity.

### 2.4. Addressing Limitations and Constraints

Stacking and hierarchy as an explicit method of design and control is in its infancy in the mechanical domain, and particularly in soft robotics, and as such the perceived limitations and benefits of this method are subject to change as progress is made in the field.

Currently, the major limitations of this method are centered on one’s ability to accurately define a module, and then the ability to combine these modules by a methodology that would be defined under ‘stacking’.

Consider two examples: (1) if the empirical relationships of blocks cannot be drawn with statistical relevance, that is to say the manufacturing process is not repeatable and reliable, then the quality of the cumulative model is severely diminished, and as such the physical meaning of any operations on abstract representations is essentially irrelevant. Similarly, (2) if the models of the modules are posed in such a way that the methods of stacking cannot converge on a solution which satisfies the design criteria relative to the module representations, then the exercise fails. Consideration of these two points will likely produce new questions as and when progress is made.

The design of a system should address, identify, and define the physical interfaces, critical parameters, technology requirements, availability of technology, life-cycle, capacity for expansion, standardization considerations, and integration concerns. By only considering the constraints when implementing the technology, one adds extra or unknown constraints, limits the capabilities of the components of the system, increases the costs due to addition of extra components, creates a longer time in designing the system, and reduces functionality from the final system.

This systems approach is used in the aerospace industry and has been described extensively by Pahl and Beitz [53] when collecting the requirements and constraints of the task.

Addressing the constraints and limitations when describing the behavior of the system formalizes the technology requirements. If the requirements, constraints, and limitations are not rigorously defined then the behavior of the system is also not well defined. Definite boundaries, interfaces, and features of modules enables the stacking of functional elements to achieve a high-level behavior. Considering the constraints and limitations when describing the behavior of the system allows more functionality from the system and permits each block to be tested, designed, and revised independently.

## 3. Moving Towards More Complex Soft Robotic Systems

A system designer can describe the behavior of the system by stacking functional blocks to create more and more complex soft robotic systems. The current design heuristic of increasing capability by stacking simple units reveals that there will be practical limits in control; the one-to-one mapping of functional block to outputs from control hardware increases the redundancy of the system while simultaneously increasing the capability.

We highlight three examples of design and control of soft robotics: Wormbot, Arthrobot, and Octobot. We believe that the Wormbot and the Octobot utilize a stacking and hierarchical approach to design.

### 3.1. Wormbot

In the Wormbot [13], the objective was to design a robot capable of exploration of an unstable or hazardous environment. The robot needed the following four requirements for the task: (1) to be capable of locomotion; (2) to be capable of movement on unstable terrain, such as sand; (3) to be sufficiently inexpensive that it can be abandoned if damaged or contaminated; and (4) to be equipped with sensors and communications systems. Soft systems were chosen because of the low cost of materials, the capability of locomotion, and the opportunity for multifunctionality with communications and sensing. Due to their soft-bodied and independent actuation in adjacent muscular walls, annelids provided the biological model for inspiration. To achieve the behavior of the annelids’ peristaltic motion, the functionality of the system was broken down into linearly actuating blocks and each functional block was stacked to achieve the behavior required. By identifying the functionality, characteristics, and constraints of the system, the blocks were designed and developed to achieve communication and linear actuation functionality. In addition, due to the modular design approach and the functionality identification, robots of any length could be quickly and easily assembled and the combinations of simple units led to the emergence of complex behaviors.

### 3.2. Arthrobot

Arthrobots are made of arachnid-inspired joints and create complex motion by actuating several of such joints [29]. An Arthrobot is a combination of several simple functional blocks, whereas a functional block is defined as an entity with a single function. The Arthrobot consists of a homogenous collective of functional blocks. Their functional block is a single joint with bending motion. When two of such blocks (joints) are stacked together, a more complex motion can be observed. If you stack even more blocks, you can build Arthrobots with *n*-legs: in their publication the authors demonstrated *n* = 6 and *n* = 8 legged Arthrobots. In general, the more blocks they stacked, the more functionality their Arthrobot acquired. The Arthrobot was designed using a bottom-up approach, stacking functional blocks to create an emergent behavior.

### 3.3. Octobot

Octobot is a fully integrated design and fabrication strategy for entirely soft autonomous robots [15]. This untethered, pneumatic robot uses a monopropellant decomposition regulated to an actuator through an embedded microfluidic logic controller. This system-level architecture is represented as an electrical analogy: check valves as diodes, fuel tanks as supply capacitors, reaction chambers as amplifiers, actuators as capacitors, vent orifices as pull-down resistors. The behavior of the Octobot was to create a complex motion through the alternate oscillation of the two groups of actuators.

To achieve this desired behavior, the control system was divided into four sections: upstream, oscillator, reaction chamber, and downstream. The electrical analogy provided an existing framework for design and the architecture was arranged to provide two functional blocks that alternated through a controller. The authors varied flowrates, tuned wall thicknesses, changed outlet diameters, and iterated through more than 30 designs and nearly 300 Octobots to converge on the suitable system-level architecture.

The Octobot is of particular interest as it represents the intersection between robotics and fluidic control. The electronic analogy provided a quick design basis for the decomposition of the behavior. We present the functional block as a combination of upstream through to downstream, to actuate the legs of the robot. The rapid fabrication process allowed the designers to make adjustments to the geometry of the robot. Although the theoretical predicted model did not match the exact operations of the Octobot, the authors addressed this with future work to the fluidic controller, reducing impedances and improving decaying clock times.

## 4. Conclusions and Future Perspectives

Soft robots have shown an increase in a diverse range of applications including subsea manipulation and rehabilitative robotics. In this perspective piece, we have discussed how stacking functional blocks has been used to increase the capacity of soft systems. This stacking of functional blocks has shown potential to produce systems that are capable of a diverse range of complex motions. The one-to-one mapping of outputs from control hardware to functional blocks has increased the capability of soft robots.

The current design heuristic of increasing capability by stacking simple units reveals that there will be practical limits in control. We predict that we will see increased amounts of autonomy in soft robotics with a trend to moving towards less control lines, but with more functional blocks. The intersection between robotics and fluidic controls, seen in the Octobot [15], is of extreme importance as the combination of control and flow-path could allow for this shift in the control paradigm. We will need to develop design tools and control paradigms that allow us to handle the complexity in these stacked, nonlinear systems.

The relationship between inputs and outputs of functional blocks must satisfy the conservation laws of mass and energy. A continuity equation can ensure that thermodynamic laws are satisfied for the design and control of soft system. An energy-flow approach, combined with a top-down engineering approach to design and control, could provide the needed tools for more complex stacked systems.

## Figures and Tables

**Table 1:** Parameters used for the construction of Figure 1.

Label ^1^	Number of Functional Blocks	Functional Block	Number of Outputs from Control Hardware	Type of Actuation	Reference
**A**	1	Fluid electrode dielectric elastomer actuators (FEDEA)	1	Dielectric elastomer	[43]
**B**	1	Expansion bladder	1	Chemical	[11]
**C**	1	Fluid-driven origami-inspired artificial muscles (FOAM)	1	Hydraulic	[44]
**D**	1	One bubble	1	Pneumatic	[38]
**E**	1	Anchoring module	1	Pneumatic	[45]
**F**	1	The arm	2	Cables and shape-memory alloy (SMA)	[8]
**G**	1	One leg	2	Pneumatic	[46]
**H**	2	Left/right chamber	2	Hydraulic	[23]
**I**	2	Cluster of four legs	2	Chemical	[15]
**J**	3	The stacked hydraulically amplified self-healing electrostatic (HASEL) actuator	1	Electrohydraulic	[7]
**K**	3	Three-unit Peano-HASEL actuator	1	Electrohydraulic	[39]
**L**	3	One vacuum-powered soft pneumatic actuator (V-SPA)	1	Pneumatic	[31]
**M**	4	Pneumatic/explosive actuator	4	Pneumatic/chemical	[47]
**N**	4	One fast pneu-net	4	Pneumatic	[48]
**O**	4	One segment	24	Pneumatic	[12]
**P**	5	One segment	1	Electromagnetic	[13]
**Q**	5	One pneu-net	5	Pneumatic	[3]
**R**	6	One pneu-net	6	Pneumatic	[1]
**S**	20	One multifilament muscle	20	Pneumatic	[41]
**T**	24	Spider-inspired joint	24	Pneumatic	[29]

^1^ The lettering on the left of the table cross-references Figure 1 and Figure 2.
